# Assessing Patient Priorities and Satisfaction Regarding Aesthetic Outcomes Following Autologous Breast Reconstruction

**DOI:** 10.1007/s00266-026-05630-8

**Published:** 2026-02-10

**Authors:** Salman Khan, Malia Voytik, Ayaka N. Deguchi, Margaret M. Hornick, Robyn B. Broach, Jessica F. Rose

**Affiliations:** 1 Division of Plastic Surgery, Department of Surgery, University of Pennsylvania Health System, Philadelphia, PA USA; 2 Perelman Center for Advanced Medicine, Clinical Surgery University of Pennsylvania, 3400 Civic Center Blvd, 14th Floor South Building, Philadelphia, PA 19104 USA

**Keywords:** Patient-reported outcomes, Autologous breast reconstruction, Aesthetics, Breast

## Abstract

**Background:**

Breast reconstruction aims to enhance patient quality of life by achieving optimal aesthetic outcomes. This study examines aesthetic preferences and satisfaction among patients undergoing autologous breast reconstruction (ABR).

**Methods:**

A 35-item survey was distributed to adult patients who underwent ABR from 2017 to 2023 at the University of Pennsylvania Health System. Patients completed three separate ranking questions in which they were asked to prioritize individual components of the breast (ranked 1–8), abdominal donor site (1–7), and nipple–areolar complex (1–6), with lower values indicating greater importance. Satisfaction was assessed using a 5-point Likert scale. Non-responders were contacted at 1 and 4 weeks, followed by phone outreach.

**Results:**

Among 301 eligible patients, 156 responded (52.1%). Breast symmetry was the highest priority (mean = 1.9, SD = 1.2), followed by shape/contour (2.6, 1.4) and position (3.1, 1.6), while NAC (5.3, 1.8) and donor site aesthetics (5.7, 1.6) ranked lowest. Satisfaction was higher for breast aesthetics (66%) than for donor site aesthetics (58%). Black patients prioritized shape/contour (64%) over symmetry (10%), while White patients prioritized symmetry (51%) over shape (26%) (*p *< 0.05). Black patients also reported greater dissatisfaction with breast (*p *= 0.006) and donor site (*p *= 0.029) aesthetics.

**Conclusion:**

This study shows that patients value different facets of their reconstruction differently. Furthermore, racial differences in aesthetic preferences and satisfaction following ABR emerged, which providers need to be mindful of. Addressing patient expectations through improved communication during the preoperative consultation may enhance aesthetic outcomes and patient satisfaction.

**Level of Evidence V:**

This journal requires that authors assign a level of evidence to each article. For a full description of these Evidence-Based Medicine ratings, please refer to the Table of Contents or the online Instructions to Authors www.springer.com/00266.

**Supplementary Information:**

The online version contains supplementary material available at 10.1007/s00266-026-05630-8.

## Introduction

Breast cancer is the most common cancer among women [1]. Over the past two decades, advancements in oncologic care have significantly improved survival rates for breast cancer patients, and an increasing number of women are opting for mastectomy over breast-conserving therapy [2]. Among post-mastectomy reconstructions, approximately one-third involve autologous tissue-based reconstruction (ABR), while the remaining two-thirds are implant-based [3]. ABR has been shown to improve patient satisfaction and quality of life, particularly in comparison with flat closure or implant-based reconstruction [4–7].

The BREAST-Q has emerged as a comprehensive tool for measuring patient satisfaction following breast surgery, encompassing scales such as satisfaction with breasts, outcome satisfaction, psychosocial well-being, sexual well-being, physical well-being, and chest/upper body satisfaction [8]. As healthcare increasingly adopts patient satisfaction as a measure of success, the BREAST-Q has gained prominence. While incredibly valuable, it does not delineate which specific aesthetic components of reconstruction are most important to each patient, necessitating further parsing to better understand individual priorities.

Achieving ideal aesthetic outcomes of ABR involves multiple features comprised of the breast, nipple–areolar complex (NAC), and donor site. While several studies have sought to define the “ideal breast,” this remains a complex concept. Bekisz et al. identified characteristics such as a moderate size, a convex lower pole, and an upper-to-lower pole volume ratio of approximately 55:45 [9]. Mallucci and Branford highlighted a 45:55 upper-to-lower pole height ratio, a slightly upward-pointing nipple, and a convex lower pole [10]. Additionally, literature suggests that patients undergoing nipple-sparing mastectomies report better quality of life, underscoring the importance of the nipple in overall outcomes [11].

The interplay of these factors, however, remains intricate and warrants further exploration. While previous studies have focused broadly on patient satisfaction and quality of life following breast reconstruction, few have systematically assessed patient-specific aesthetic priorities across different features of the reconstructed breast, donor site, and NAC. Moreover, little is known about how demographic differences such as race, age, revisions, or NAC retention, influence these priorities and specific satisfaction outcomes. By directly evaluating patient-reported aesthetic priorities and satisfaction with individual reconstructive components, this study aims to better understand patient reconstructive goals following ABR, allowing for more individualized surgical planning and preoperative counseling.

## Patients and Methods

Following Institutional Review Board approval (IRB # 856894) an anonymous survey was sent to patients who received abdominal free flap ABR following either nipple sparing or skin sparing mastectomy at the University of Pennsylvania Health System from January of 2017 to August 2023. The survey instrument was designed using the online survey tool Qualtrics. The 10-minute, 35-item purpose-designed survey was developed to assess breast, nipple–areolar complex, and donor site aesthetic priorities not captured in existing validated instruments such as the BREAST-Q (Appendix 1). Content validity was supported through expert review by two plastic surgeons specializing in autologous reconstruction, who refined items for clarity and relevance through an iterative process. Patients completed three separate ranking questions in which they were asked to prioritize individual components of the breast (ranked 1–8), abdominal donor site (1–7), and nipple–areolar complex (1–6), with lower values indicating greater importance. A 5-point Likert scale was used to assess satisfaction of individual components of the breast and donor site, as well as overall satisfaction.

Non-responders were emailed reminders to participate at 1 and 4 weeks after initial invite, then called at 6–8 weeks. Most respondents were at least one year post-reconstruction, ensuring outcomes were relatively stable. Statistical analysis was performed using R 4.4.0 (Rcorp, Vienna Austria).

## Results

### Demographics

A total of 301 patients were invited to participate in the survey, and 156 (52.1%) responded. The average patient age was 55 years (SD 10). Most identified as White (80%), while 8.6% identified as Black, and 12.4% identified as another race. The majority of patients underwent at least one revision with most undergoing 1–2 (63%). Demographic characteristics are summarized in Table 1.

**Table 1:** Patient demographics

Variable	*N* = 155^1^
Age	55 (10)
BMI	28.5 (5.6)
*Race*	
Black	13/152 (8.6%)
White More than one race	121/152 (8.6%) 11/152 (7.2%)
Asian	7/152 (4.6%)
Years since surgery	
0–1	3/153 (2%)
1–3	88/153 (92%)
3–5	57/153 (37.3%)
5+	5/153 (3.3%)
*Did you and your plastic surgeon discuss your aesthetic goals prior to reconstruction?*	
No	12/153 (7.8%)
Yes	141/153 (92%)
*Did you retain your native NAC*	
Yes	41/142 (19&)
No	101/142 (71%)
*Number of revisions*	
0	39/136 (29%)
1–2	86/163 (63%)
3–4	8/136 (5.9%)
4+	3/136 (2.2%)

### Aesthetic Priorities

**Fig. 1:**
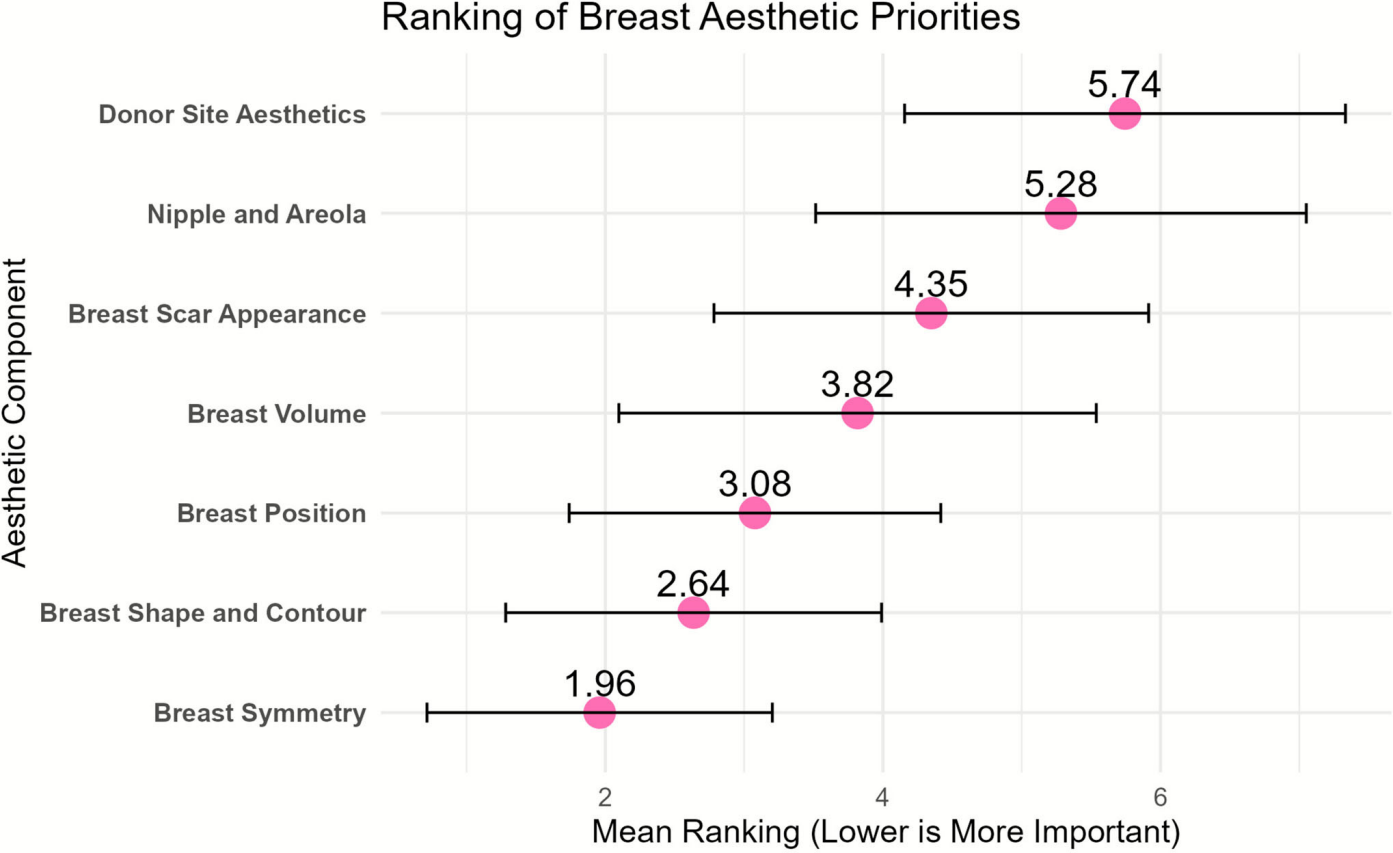
Ranking of priorities with regards to breast aesthetic components

**Fig. 2:**
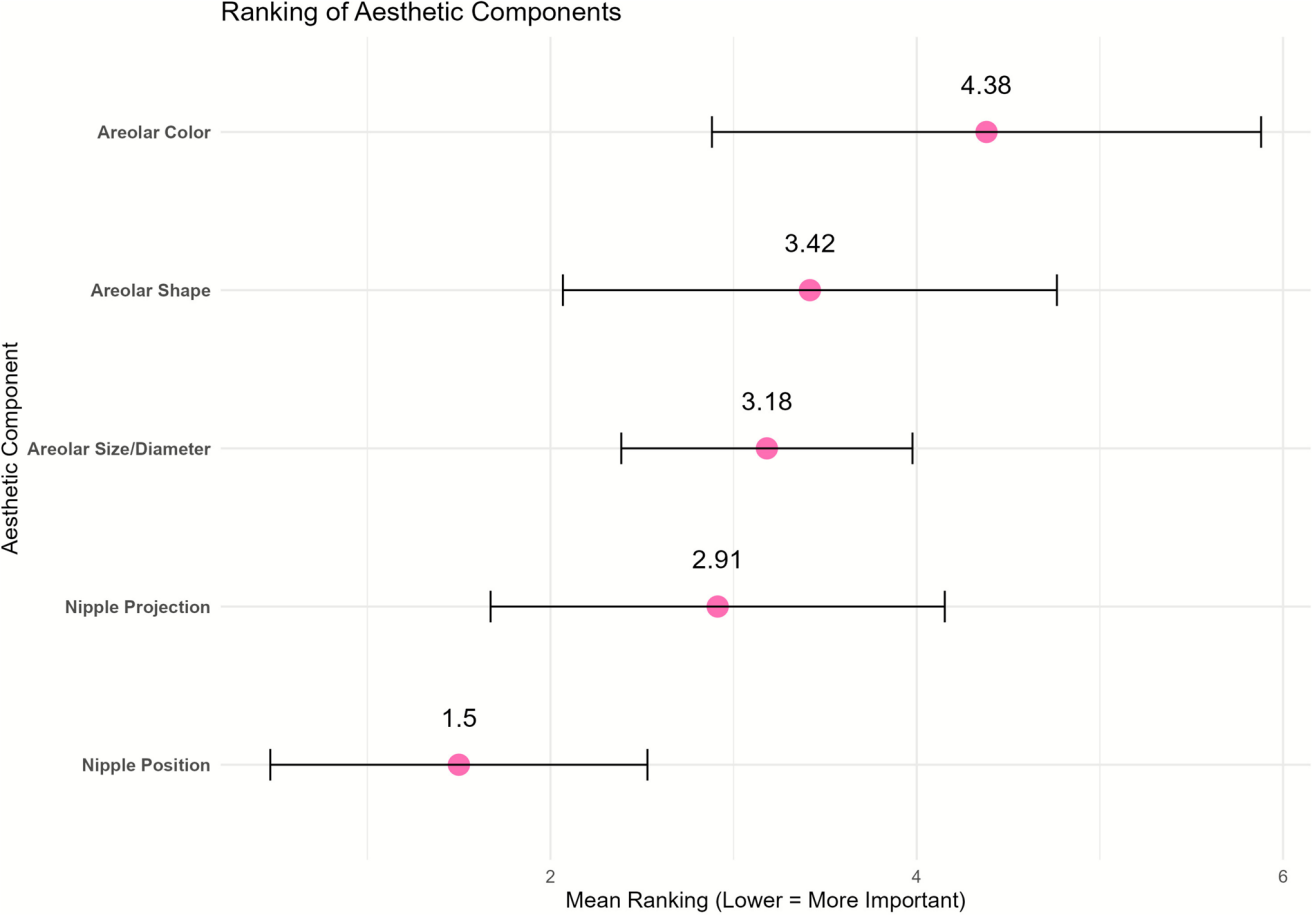
Ranking of priorities with regards to NAC components

**Fig. 3:**
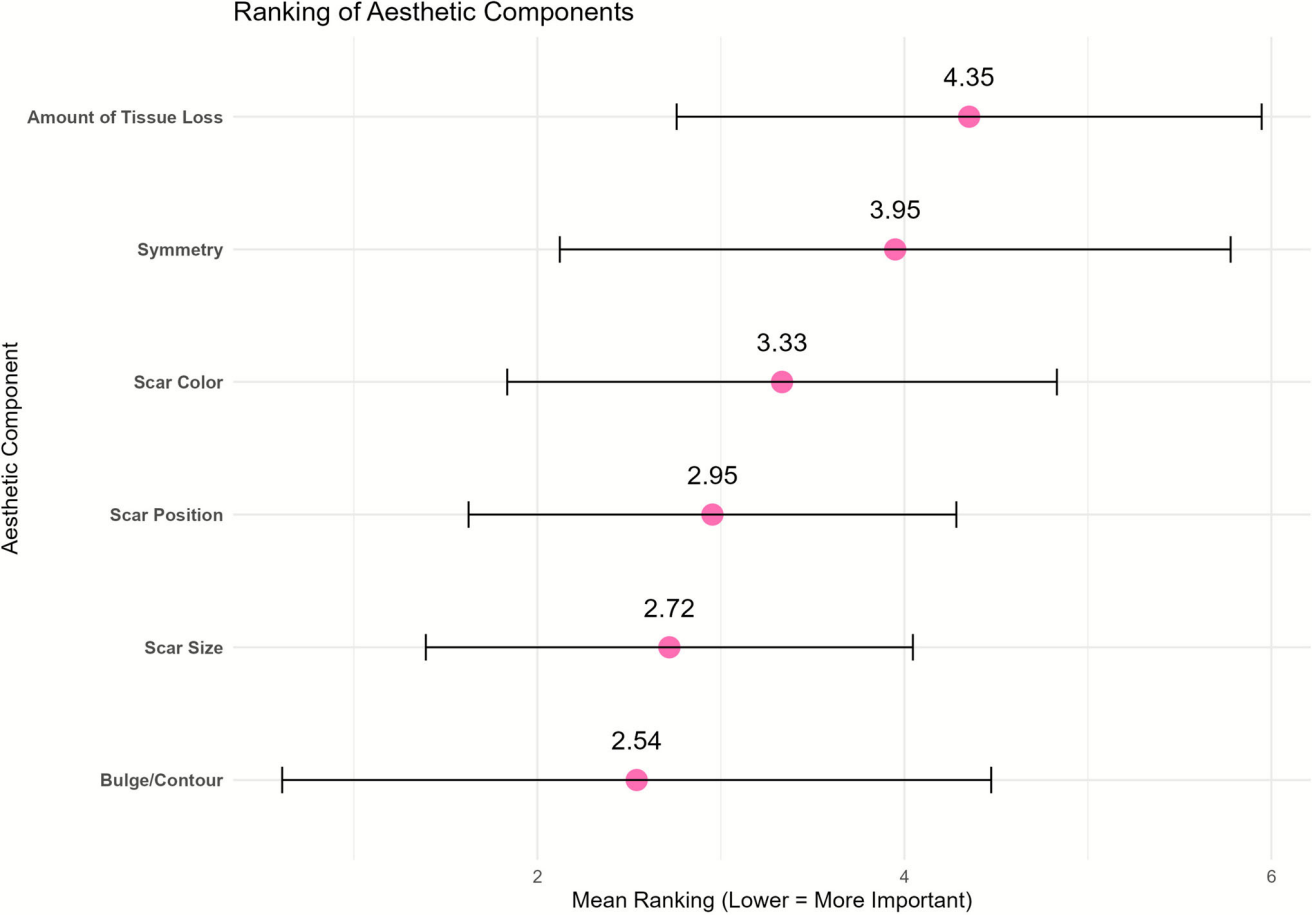
Ranking of priorities with regards to donor site aesthetic components

Breast symmetry was the top priority (mean = 1.9, SD = 1.2), followed by shape/contour (2.64, 1.35) and breast position (3.07, 1.6), while NAC (5.3, 1.8) and donor site aesthetics (5.7, 1.6) ranked lowest (Fig. 1). Among those who retained their NAC, nipple position (1.5, 1.0) was rated most important, while areolar color (4.3, 1.5) was least important (Fig. 2). For donor site aesthetics, bulge/contour (2.5, 1.9) and scar size (2.7, 1.3) were prioritized over tissue loss (4.3, 1.6) (Fig. 3).

### Satisfaction

The majority of patients were somewhat or very satisfied with their breast appearance (66%) and donor site appearance (58%). Satisfaction was highest for breast symmetry (76%) and position (79%) and lowest for NAC aesthetics (48%) and donor site scar components, including scar color (53%) and scar size (47%).

### Nipple Areolar Complex Retention

Among patients who lost their NAC (71%), 73% reported that losing their NAC was moderately to not important for their aesthetic outcome, while 9.2% considered losing it extremely important, and 18% regarded it as very important. Patients who retained their NAC were more likely to report satisfaction with their breasts (86%) compared to those who did not (77%), although this difference was not statistically significant (*p = *0.30). There were also no significant differences between groups in perceived attractiveness or confidence (*p *> 0.05).

In terms of breast component aesthetic priorities, patients who lost their NAC rated it as significantly less important (*p *= 0.024) than those who retained their native NAC, with a mean ranking of 5.51 (SD 1.69) compared to 4.68 (SD 1.87). However, patients who lost their NAC ranked breast shape and contour as significantly more important (2.48 vs. 3.13, *p *= 0.012).

### Racial Differences

**Fig. 4:**
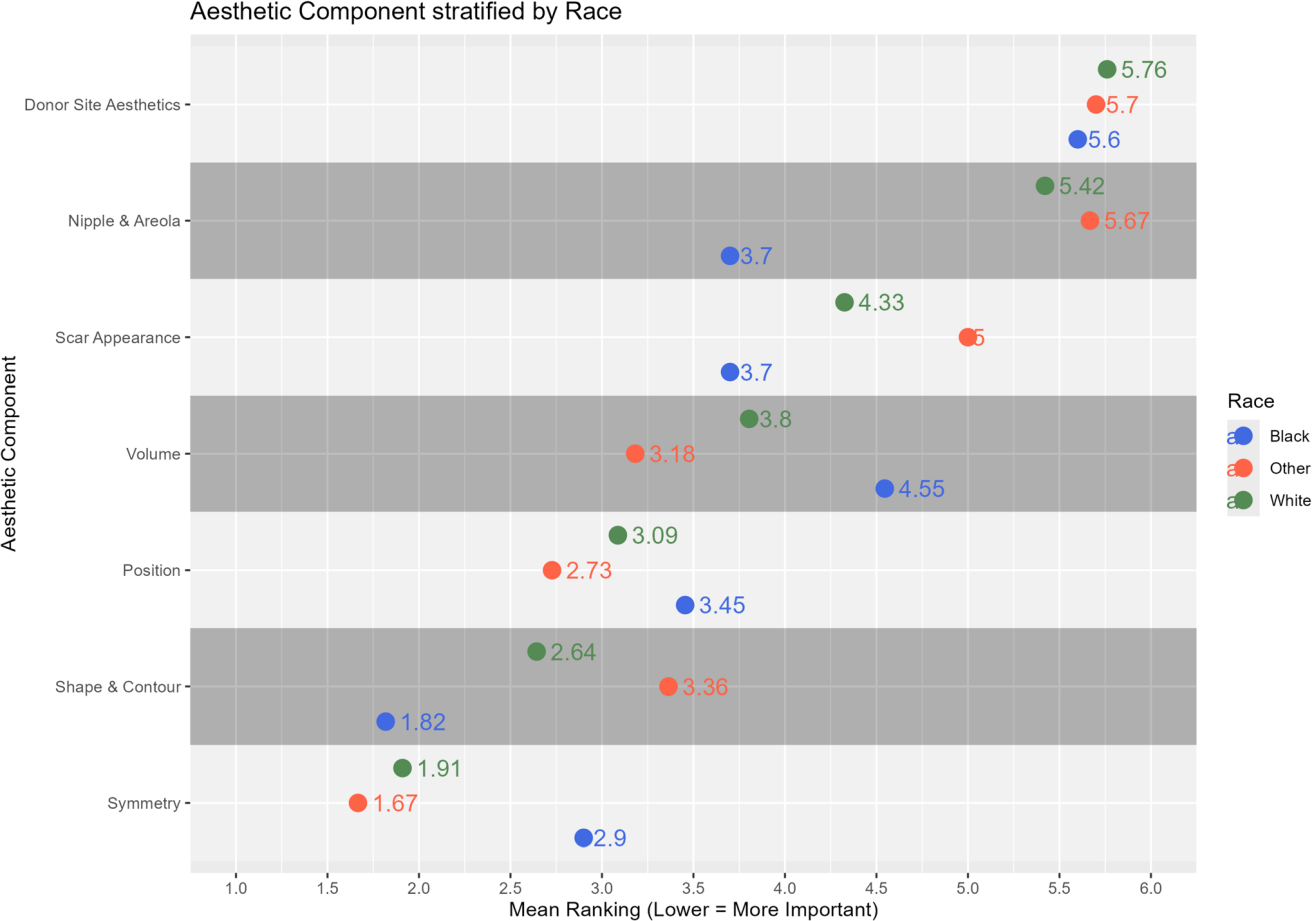
Ranking of priorities with regards to breast aesthetic components stratified by race

**Fig. 5:**
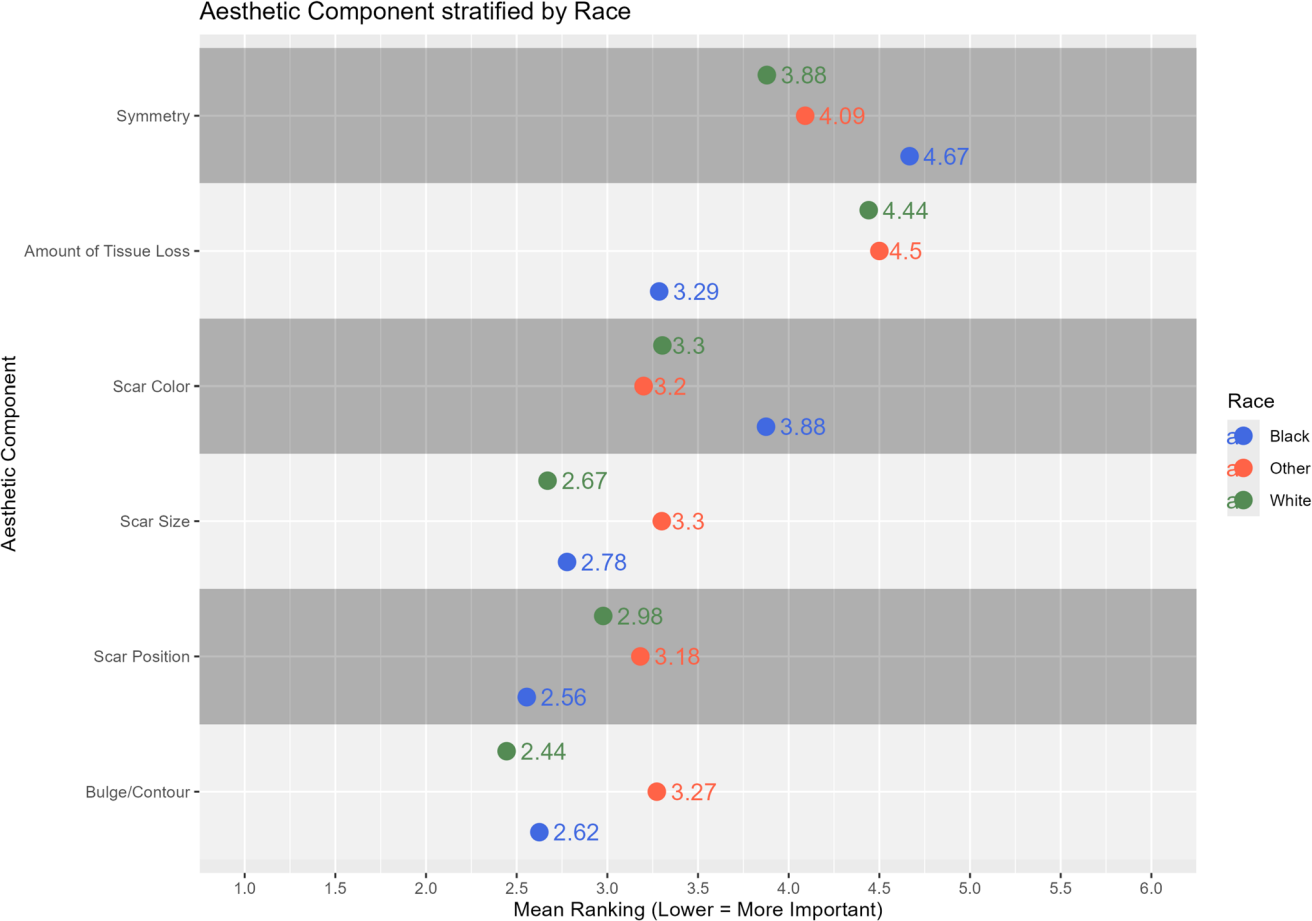
Ranking of priorities with regards to donor site aesthetic components stratified by race

Black patients had a higher BMI than patients of other races but were otherwise comparable (Table 2). Breast symmetry was the top aesthetic priority for 51% of White patients, but only for 10% of Black patients (*p *< 0.05). In contrast, 64% of Black patients ranked breast shape and contour as their highest priority, compared to 26% of White patients (*p *< 0.05) (Fig. 4). No significant differences were observed in donor site priorities (Fig. 5).

**Table 2:** Patient responses by race

Characteristic	Other *N* = 18^1^	Black *N* = 13^1^	White *N* = 121^1^	*p*-value^2^
Age	55 (47, 63)	56 (54, 63)	55 (47, 63)	0.7
BMI	23.8 (22.5, 30.8)	33.3 (29.1, 36.6)	27.4 (24.8, 30.8)	0.002
*Were reconstruction goals discussed?*				>0.9
No	1 (5.6%)	1 (7.7%)	10 (8.3%)	
Yes	17 (94%)	12 (92%)	111 (92%)	
*Native NAC retained?*				0.9
Yes	5 (31%)	3 (23%)	33 (29%)	
No	11 (69%)	10 (77%)	79 (71%)	
*Satisfaction with breast reconstruction appearance*				0.006
Strongly dissatisfied	1 (7.1%)	4 (33%)	6 (5.5%)	
Somewhat dissatisfied	0 (0%)	2 (17%)	13 (12%)	
Neither satisfied nor dissatisfied	2 (14%)	1 (8.3%)	5 (4.5%)	
Somewhat satisfied	3 (21%)	0 (0%)	44 (40%)	
Strongly satisfied	8 (57%)	5 (42%)	42 (38%)	
*Satisfaction with donor site appearance*				0.029
Strongly dissatisfied	1 (7.1%)	3 (25%)	8 (7.3%)	
Somewhat dissatisfied	1 (7.1%)	3 (25%)	20 (18%)	
Neither satisfied nor dissatisfied	1 (7.1%)	0 (0%)	8 (7.3%)	
Somewhat satisfied	9 (64%)	0 (0%)	50 (46%)	
Strongly satisfied	2 (14%)	6 (50%)	23 (21%)	
*My overall aesthetic results matched my expectation*				0.022
Strongly disagree	3 (21%)	3 (25%)	6 (5.5%)	
Somewhat disagree	0 (0%)	2 (17%)	18 (16%)	
Neither agree nor disagree	2 (14%)	1 (8.3%)	9 (8.2%)	
Somewhat agree	6 (43%)	0 (0%)	48 (44%)	
Strongly agree	3 (21%)	6 (50%)	29 (26%)	
*I am more confident with breast reconstruction than I would have been without*				0.055
Strongly disagree	1 (7.1%)	3 (25%)	5 (4.5%)	
Somewhat disagree	1 (7.1%)	0 (0%)	5 (4.5%)	
Neither agree nor disagree	3 (21%)	1 (8.3%)	6 (5.5%)	
Somewhat agree	0 (0%)	0 (0%)	13 (12%)	
Strongly agree	9 (64%)	8 (67%)	81 (74%)	
*I have decreased feelings of attractiveness because of my choice in breast reconstruction*				0.2
Strongly disagree	6 (43%)	6 (50%)	33 (30%)	
Somewhat disagree	2 (14%)	1 (8.3%)	16 (15%)	
Neither agree nor disagree	4 (29%)	0 (0%)	22 (20%)	
Somewhat agree	2 (14%)	2 (17%)	28 (26%)	
Strongly agree	0 (0%)	3 (25%)	10 (9.2%)	
*Number of revisions*				0.3
0	5 (36%)	2 (17%)	32 (29%)	
1–2	9 (64%)	9 (75%)	68 (62%)	
2–3	0 (0%)	0 (0%)	8 (7.3%)	
3+	0 (0%)	1 (8.3%)	1 (0.9%)	
*Did your revisions improve your aesthetic outcome?*				0.3
Yes	8 (89%)	6 (60%)	59 (79%)	
No	1 (11%)	4 (40%)	16 (21%)	
*Was the surgical/recovery burden of autologous breast reconstructions and the subsequent revisions worth the improvement in your overall feelings about your appearance*				0.059
1	9 (69%)	8 (67%)	91 (88%)	
2	4 (31%)	4 (33%)	13 (13%)	

^1^Median (Q1, Q3); *n* (%)

^2^Kruskal-Wallis rank sum test; Pearson's Chi-squared test

Black patients were more likely to report dissatisfaction with both breast (*p *= 0.006) and donor site (*p *= 0.029) aesthetics. Dissatisfaction with breast scar appearance was reported by 33.3% of Black patients compared to 22.7% of White patients (*p *= 0.11). Extreme dissatisfaction with donor site scar color was reported by 25% of Black patients versus 6.6% of White patients (*p *= 0.05), while extreme dissatisfaction with donor site scar size was reported by 17% of Black patients compared to 6.6% of White patients (*p *= 0.20).

### Revisions, Age, BMI, Time Since Surgery

Aesthetic priorities, patient satisfaction, and revision rates were stratified by BMI categories, age, and time since surgery with no significant differences. There was also no significant difference in patient satisfaction rates or aesthetic preference by revision rates or between patients who did and did not undergo a revision (all *p *> 0.05).

## Discussion

This anonymous, survey-based study elucidated distinct patient perspectives regarding both satisfaction and priorities in autologous breast reconstruction. To our knowledge, it is the first to examine the importance of the individual components of reconstruction and demonstrate their potential influence on patient satisfaction. As the number of patients undergoing breast reconstruction grows, understanding what constitutes an “aesthetic breast,” and recognizing that this perception varies among individuals, becomes increasingly important.

In this study, patients identified breast symmetry as the top priority, followed by breast position and shape/contour. These findings support earlier work emphasizing that symmetry is central to overall aesthetic satisfaction [12–14]. In a study of patients undergoing reconstruction, vertical extent asymmetry led to higher dissatisfaction [12]. This correlates with literature suggesting that the upper: lower pole ratio is a key determinant of an aesthetically pleasing breast [9, 10]. Barone et al. demonstrated that managing contralateral asymmetry after implant-based reconstruction significantly improved patient satisfaction [15]. Flap choice (e.g., TRAM vs. DIEP) does not appear to impact symmetry, as Cohen et al. found no difference between these two approaches [16]. Breast position and shape are interrelated with symmetry, and these factors must be addressed with consideration of patient BMI, especially in unilateral reconstructions where contralateral matching is critical [15, 17].

Overall, the NAC did not emerge as a high priority for our surveyed population, although this may be due to recall bias as most patients had lost their nipple during mastectomy. This is suggested by the fact that patients without an NAC ranked it as of lower importance. However, even those who did retain their NAC ranked it as less important on average than breast symmetry, position, shape and contour, and volume. This may explain why we observed no difference in breast satisfaction between patients who retained their NAC and those who did not. This contrasts with prior literature, which found that nipple-sparing mastectomy or NAC reconstruction often results in higher patient satisfaction [18, 19]. When reconstructing or maintaining the NAC, this study suggests focusing on position, projection, and diameter. Importantly, reconstructive approaches in our institution are relatively uniform, limited to nipple-sparing or skin-sparing mastectomy, ensuring comparability.

Similarly, breast revisions were not associated with improved satisfaction. This finding supports a recent study which, through multivariate analysis of patients following breast reconstruction, found that neither breast revision nor NAC reconstruction was significantly associated with improved 1-year Satisfaction with Breasts scores [20].

Our study also identified racial differences in satisfaction and priorities. Black patients were more likely to report dissatisfaction with both breast and donor site aesthetics, which is consistent with existing literature [21]. This finding is especially relevant given that Black patients were more likely to prioritize shape/contour over symmetry. Much of the existing literature on the ideal breast emphasizes symmetry, which may not fully reflect the aesthetic values of all patient populations. Although limited by small subgroup size, these findings are consistent with prior research demonstrating race-based variations in breast aesthetic preferences [22, 23].

Although differences in scar satisfaction did not reach statistical significance, Black patients in our cohort reported higher rates of extreme dissatisfaction with both breast and donor site scars. This trend aligns with prior literature reporting that Black patients often experience more persistent scar-related symptoms and have higher incidences of hypertrophic scarring [24, 25]. While our study was not powered to fully explore these differences, they underscore the importance of explicitly discussing scar aesthetics and management in preoperative consultations. Tailored strategies, such as early counseling and scar-specific interventions, may help address patients’ expectations and improve satisfaction across diverse populations [26–29].

Dissatisfaction with the donor site was a common concern in our cohort, reflecting the conclusions of a systematic review that highlighted a general lack of attention to donor site aesthetics in the past decade [30]. Participants who were unhappy with their donor sites also tended to express higher dissatisfaction with overall aesthetics, possibly because suboptimal donor site results can overshadow the final reconstructive outcome. Common causes of donor site dissatisfaction after DIEP flap reconstruction include bulging or “standing cone” deformities, residual abdominal overhang, uneven abdominal wall thickness above and below the scar, and the scar’s location [31]. Our study confirmed these points, with bulge/contour issues ranking highest, followed by scar size, position, and color. Wagner et al. proposed a low DIEP flap design, if a dominant perforator is present, to place the scar nearer the pelvic rim, improving concealment. Similarly, plication of rectus diastasis with nonabsorbable sutures can enhance abdominal contour and prevent bulging. Progressive tension sutures (often barbed) can further minimize closure tension, obliterate dead space, and reduce wound dehiscence or scar widening, ultimately yielding a more aesthetic donor site scar [32].

Despite the novel insights provided in this study, it was limited by its retrospective nature and anonymous survey design, which prevented detailed patient follow-up, or correlation with surgical details and outcomes. However, this design was chosen to ensure the integrity of patient response. Additionally, the survey instrument used in this study was not formally validated, which may limit generalizability. However, it was developed through expert review and informed by established aesthetic satisfaction constructs. Future research should include formal psychometric validation of this instrument and evaluation across diverse patient populations. Furthermore, the limited sample size reduced the ability to perform further subgroup analysis. Prior literature suggests that patients of Asian descent may have lower satisfaction and quality-of-life scores post-reconstruction [20]. Our study included only seven Asian patients, necessitating their inclusion in a broader ‘other’ group due to limited power. Previous research has proposed that lower satisfaction in Asian populations might stem from linguistic and cultural barriers, or lower health literacy [21, 33]. It would be valuable to assess the aesthetic priorities of Asian patients and other minority groups in future investigations to clarify how their concerns may differ.

Future prospective studies should evaluate aesthetic priorities before and after reconstruction to assess how perceptions change over time. Additionally, studies incorporating objective scar assessment and long-term follow-up may help further clarify factors influencing satisfaction.

By better understanding patient priorities and refining preoperative counseling, providers can ensure more personalized surgical planning and improved patient satisfaction. Addressing both reported and overlooked concerns will be essential in optimizing outcomes in breast reconstruction.

## Conclusion

This study highlights the multifaceted nature of aesthetic satisfaction in ABR. By examining priorities for the breast, nipple, and donor site, we have shown that factors such as symmetry, shape/contour, and scar appearance significantly influence patients’ overall satisfaction. Notably, racial differences in priorities and scar-related dissatisfaction emphasize the need for individualized approaches in surgical planning and patient counseling. Although the retrospective design and survey-based method limit direct correlation with surgical outcomes, these findings underscore the importance of customized preoperative counseling. Moving forward, prospective, long-term studies incorporating broader demographic inclusion will be crucial for refining patient-centered care and optimizing reconstructive outcomes.

## Supplementary Information

Below is the link to the electronic supplementary material.

Supplementary file1 (DOCX 24 kb)
