# Supplementary material for: Assessing Patient Priorities and Satisfaction Regarding Aesthetic Outcomes Following Autologous Breast Reconstruction
Source: Aesthetic Plast Surg. 2026 Feb 10;50(11):3925–34. doi: 10.1007/s00266-026-05630-8 (PMC13315328; doi:10.1007/s00266-026-05630-8)
Supplement: Supplementary file 1 — Supplementary file1 (DOCX 24 kb) [file 266_2026_5630_MOESM1_ESM.docx]

BRA patient survey

Q2.1 How old are you?

________________________________________________________________

Q2.2 What is your height?

|  | (1) |
| --- | --- |
| Feet (1) |  |
| Inches (2) |  |

Q2.3 What is your weight (lbs)?

________________________________________________________________

Q2.4 What is your race/ethnicity? (select all that apply)

- American Indian or Alaska Native (1)
- Asian (2)
- Black or African American (3)
- Hispanic or Latino (4)
- Middle Eastern or North African (5)
- Native Hawaiian or Pacific Islander (6)
- White (7)
- Other (FIll in the BLank) (8) __________________________________________________

Q2.5 How many years ago did you have your autologous breast reconstruction?

- 0-1 years (1)
- 1-3 years (2)
- 3-5 years (3)
- 5+ years (4)

Q2.6 Did you and your plastic surgeon discuss your aesthetic goals prior to reconstruction?

- No (1)
- Yes (2)

Q2.7 What are your aesthetic/appearance priorities with regards to your breasts following reconstruction? (Drag and rank in order of importance of to you)

______ Breast Symmetry (1)

______ Breast Position (2)

______ Breast Volume (3)

______ Breast Shape and Contour (4)

______ Scar Appearance (5)

______ Nipple and Areola (area immediately surrounding the nipple) (6)

______ Donor Site Aesthetic (7)

______ Other (8)

Q2.8 Did you retain your nipple and areola following mastectomy?

- Yes (1)
- No (2)

Display This Question:

If Q2.8 = 1

Q2.9 What are your aesthetic priorities with regards to your nipples following reconstructive surgery? (Drag and rank in order of importance of to you)

______ Nipple Projection (how much it protrudes from your breast) (1)

______ Nipple position (2)

______ Areolar size/diameter (3)

______ Areolar shape (4)

______ Areolar color (5)

______ Other (fill in the blank) (6)

Display This Question:

If Q2.8 = 2

Q2.10 How significant is losing your nipples to your overall aesthetic outcome?

- Not at all important (1)
- Slightly important (2)
- Moderately important (3)
- Very important (4)
- Extremely important (5)

Q2.11 With your donor site (abdomen) in mind, what is aesthetically important? (Drag and rank in order of importance of to you)

______ Scar Color (1)

______ Scar Size (2)

______ Scar position (3)

______ Amount of tissue loss (4)

______ Symmetry (5)

______ Bulge/contour of your donor site (6)

______ Other (7)

Q2.12 Please select how strongly you agree with the following statement
 I am satisfied with my breast reconstruction appearance.

- Strongly disagree (1)
- Somewhat disagree (2)
- Neither agree nor disagree (3)
- Somewhat agree (4)
- Strongly agree (5)

Q2.13 Please select how strongly you agree with the following statement
 I am satisfied with my donor site appearance.

- Strongly disagree (1)
- Somewhat disagree (2)
- Neither agree nor disagree (3)
- Somewhat agree (4)
- Strongly agree (5)

Q2.14 Please select how strongly you agree with the following statement
 My overall aesthetic results matched my expectation.

- Strongly disagree (1)
- Somewhat disagree (2)
- Neither agree nor disagree (3)
- Somewhat agree (4)
- Strongly agree (5)

Q2.15 Please select how strongly you agree with the following statement
 I am more confident with breast reconstruction than I would have been without.

- Strongly disagree (1)
- Somewhat disagree (2)
- Neither agree nor disagree (3)
- Somewhat agree (4)
- Strongly agree (5)

Q2.16 Please select how strongly you agree with the following statement
 I have decreased feelings of attractiveness because of my choice in breast reconstruction.

- Strongly disagree (1)
- Somewhat disagree (2)
- Neither agree nor disagree (3)
- Somewhat agree (4)
- Strongly agree (5)

Q2.17 How many revisions have you had?

- 0 (1)
- 1-2 (2)
- 3-4 (3)
- 4+ (4)

Display This Question:

If Q2.17 != 1

Q2.18 Did your revisions improve your aesthetic outcome?

- Yes (1)
- No (2)

Q2.19 Was the surgical/recovery burden of autologous breast reconstructions and the subsequent revisions worth the improvement in your overall feelings about your appearance?

- Yes (1)
- No (2)

Q2.20 How satisfied are you with these aspects of your breast reconstruction?

|  | Extremely dissatisfied (1) | Somewhat dissatisfied (2) | Neither satisfied nor dissatisfied (3) | Somewhat satisfied (4) | Extremely satisfied (5) |
| --- | --- | --- | --- | --- | --- |
| Breast Symmetry (1) |  |  |  |  |  |
| Breast Position (2) |  |  |  |  |  |
| Breast Volume (3) |  |  |  |  |  |
| Breast Shape and Contour (4) |  |  |  |  |  |
| Scar Appearance (5) |  |  |  |  |  |
| Nipple Areolar Complex (area immediately surrounding the nipple) (6) |  |  |  |  |  |
| Donor Site Aesthetic (7) |  |  |  |  |  |
| Other (8) |  |  |  |  |  |

Q2.21 How satisfied are you with your donor site (abdomen)?

|  | Extremely dissatisfied (1) | Somewhat dissatisfied (2) | Neither satisfied nor dissatisfied (3) | Somewhat satisfied (4) | Extremely satisfied (5) |
| --- | --- | --- | --- | --- | --- |
| Scar Color (1) |  |  |  |  |  |
| Scar Size (2) |  |  |  |  |  |
| Scar position (3) |  |  |  |  |  |
| Amount of tissue loss (4) |  |  |  |  |  |
| Symmetry (5) |  |  |  |  |  |
| The bulge/contour of your donor site (6) |  |  |  |  |  |
| Other (7) |  |  |  |  |  |

End of Block: Default Question Block
